# Inhibition of SARS-CoV-2 Replication by Self-Assembled siRNA Nanoparticles Targeting Multiple Highly Conserved Viral Sequences

**DOI:** 10.3390/v16071072

**Published:** 2024-07-03

**Authors:** Jianan Sun, Siya Lu, Jizhen Xiao, Nuo Xu, Yingbin Li, Jinfeng Xu, Maohua Deng, Hanlu Xuanyuan, Yushi Zhang, Fangli Wu, Weibo Jin, Kuancheng Liu

**Affiliations:** 1 School of Public Health (Shenzhen), Shenzhen Campus of Sun Yat-sen University, Shenzhen 518107, China; 2 School of Public Health (Shenzhen), Sun Yat-sen University, Guangzhou 510275, China; 3 College of Life Sciences & Medicine, Zhejiang Sci-Tech University, Hangzhou 310018, China; 4 Shenzhen Key Laboratory of Pathogenic Microbes and Biosafety, Shenzhen Campus of Sun Yat-sen University, Shenzhen 518107, China

**Keywords:** SARS-CoV-2, siRNA, RNA nanotechnology, replication

## Abstract

Coronavirus infectious disease 2019 (COVID-19), caused by severe acute respiratory virus type 2 (SARS-CoV-2), has caused a global public health crisis. As an RNA virus, the high gene mutability of SARS-CoV-2 poses significant challenges to the development of broad-spectrum vaccines and antiviral therapeutics. There remains a lack of specific therapeutics directly targeting SARS-CoV-2. With the ability to efficiently inhibit the expression of target genes in a sequence-specific way, small interfering RNA (siRNA) therapy has exhibited significant potential in antiviral and other disease treatments. In this work, we presented a highly effective self-assembled siRNA nanoparticle targeting multiple highly conserved regions of SARS-CoV-2. The siRNA sequences targeting viral conserved regions were first screened and evaluated by their thermodynamic features, off-target effects, and secondary structure toxicities. RNA motifs including siRNA sequences were then designed and self-assembled into siRNA nanoparticles. These siRNA nanoparticles demonstrated remarkable uniformity and stability and efficiently entered cells directly through cellular endocytic pathways. Moreover, these nanoparticles effectively inhibited the replication of SARS-CoV-2, exhibiting a superior inhibitory effect compared to free siRNA. These results demonstrated that these self-assembled siRNA nanoparticles targeting highly conserved regions of SARS-CoV-2 represent highly effective antiviral candidates for the treatment of infections, and are promisingly effective against current and future viral variants.

## 1. Introduction

Since 2019, the global COVID-19 pandemic resulting from severe acute respiratory virus type 2 (SARS-CoV-2) infection has caused serious health, social, and economic disruptions worldwide. According to the World Health Organization (WHO), as of 31 March 2024, over 775 million people have been infected, resulting in more than 7.04 million deaths. Although vaccines and antivirals including inhibitors targeting viral proteins and monoclonal antibodies have been widely used in the prevention and treatment of COVID-19, there remains a need for additional therapeutic options. This is particularly critical due to the continual mutation and evolution of the virus, underscoring the significance of broad-spectrum antivirals targeting conserved viral components or universal host targets [1].

SARS-CoV-2 is an enveloped virus with a positive-sense single-stranded RNA genome that is approximately 30 kilobase (kb) in length. It exhibits approximately 80% genetic similarity to SARS-CoV and 50% to MERS-CoV, belonging to the family of β-coronaviruses [2,3]. The genome of SARS-CoV-2 includes 14 open reading frames (ORFs), encoding 4 structural proteins, namely the spike (S), envelope (E), membrane (M), and nucleocapsid (N) proteins, 16 non-structural proteins (NSP1-16), and 6 accessory proteins. SARS-CoV-2 enters host cells via the spike protein, mainly through attachment and fusion mechanisms. This involves the S1 subunit of the spike protein binding to the ACE receptor and the S2 subunit anchoring the S protein on the virus membrane to mediate membrane fusion [4,5]. Following fusion of the cellular and viral membranes, the nucleocapsid enters the cytoplasm and the genome is released from the N protein by host cellular proteases. The viral genome directly functions as template mRNA for the translation of ORF1a and ORF1ab to yield proteins pp1a and pp1ab, followed by the initiation of replication of the viral RNA genome and transcription of viral subgenomic RNAs. Once newly synthesized RNA genomes and structural proteins are produced, progeny virions are then formed and released [6,7].

Small interfering RNAs (siRNAs) with 2 nt 3′ overhangs mediate efficient sequence-specific mRNA degradation, exhibiting significant therapeutic potential in antiviral and other disease treatments [8]. As an RNA virus, SARS-CoV-2 is highly susceptible to RNA interference (RNAi) including siRNA [9,10]. It has been reported that SARS-CoV-2 replication could be efficiently inhibited in in vitro and in vivo studies [10,11,12,13,14]. However, the rapid emergence of new variants of concern (VOCs) of SARS-CoV-2 poses challenges for current antivirals, particularly concerning their efficacy and broad spectrum. This underscores the critical need to develop siRNAs capable of targeting both current and potential future VOCs [15,16]. Furthermore, the susceptibility of siRNA to degradation, along with concerns regarding its delivery efficiency, are also critical aspects that warrant attention in the development of siRNA therapy [8,17]. In general, siRNA can be delivered by coupling with targeting moieties or using various nanoparticle-based systems, including inorganic, lipid, and polymeric systems [8,17,18]. However, these delivery methods also have limitations, such as improper accumulation in the body, challenges related to biocompatibility, and the risk of endotoxin contamination [8]. Currently, only siRNA delivered through conjugation with GalNAc and encapsulation within lipid nanoparticles (LNPs) are approved for clinical use [19,20,21,22,23,24].

Over the past two decades, RNA has evolved from a mere genetic messenger into a multifaceted therapeutic tool. Progress in RNA nanotechnology, particularly in drug delivery and gene therapy, has unveiled new therapeutic possibilities. This technology leverages RNA’s inherent ability to self-fold and engage in Watson–Crick base pairing, forming stable nano-sized structures with specific shapes and functions. The programmability and modifiability of RNA enable the design of nanoparticles capable of autonomously assembling into complex structures carrying therapeutic agents such as siRNA, mRNA vaccines, fluorescent dyes, and proteins [25]. Moreover, by incorporating multiple siRNAs into nanoparticle design, different regions of a virus genome can be concurrently targeted, limiting the emergence of escape mutations [26]. Modifications such as 2′-fluoro and 2′-O-methyl can enhance RNA nanoparticle stability, reduce immunogenicity, and enable controlled activation, thereby improving biocompatibility and functionality [11,27,28]. These optimizations enhance performance in gene therapy and drug delivery, increasing circulation, enhancing target specificity, and minimizing immune responses [29]. Compared to other nanoparticle-based delivery systems, RNA nanoparticles are more biocompatible and less toxic. The automation of the assembly process is feasible by adjusting conditions such as temperature and salt concentration [30]. With significant potential in antiviral, cancer, and genetic disease therapies, RNA nanoparticles offer a promising strategy for advancing RNA gene therapy toward greater safety and efficacy.

In this study, we developed self-assembled siRNA nanoparticles targeting highly conserved regions of SARS-CoV-2 employing RNA nanotechnology and have demonstrated their efficient inhibitory effects on SARS-CoV-2 replication.

## 2. Materials and Methods

### 2.1. Design of siRNA Sequences

**Figure 1:**
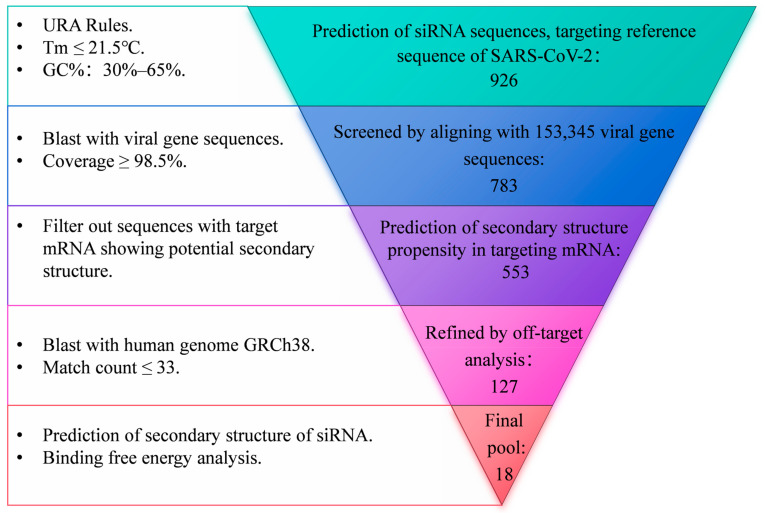
Flowchart of siRNA design.

To obtain siRNA sequences targeting the highly conserved regions of SARS-CoV-2, a systematic screen approach was employed, as illustrated in Figure 1. Briefly, an siRNA pool was first established utilizing the siDirect2.0 siRNA design tool based on the reference genome of SARS-CoV-2 (NC_045512.2). The siRNA sequences adhered to the Ui-Tei Rules [31], Reynolds Rules [32], or Amarzguioui Rules [33], ensuring that the melting temperature (Tm) of both strands was less than or equal to 21.5 °C and that the GC content ranged from 30% to 65%. All siRNA sequences were subsequently aligned with 153,345 SARS-CoV-2 genome sequences retrieved from the SARS-CoV-2 Data Hub of NCBI, spanning sample collection dates from 29 November 2019 to 31 December 2022. These sequences encompassed major variants of concern (VOCs) including Alpha (B.1.1.7), Beta (B.1.351), Gamma (P.1), Delta (B.1.617.2), and Omicron (BA.5). siRNAs exhibiting a coverage rate of over 98.5% of viral genome sequences were initially selected and subsequently refined through off-target analysis against the human genome GRCh38, ensuring that the number of predicted off-target sites was 33 or fewer. The siRNAs targeting mRNAs with a high tendency for secondary structure toxicity were excluded [34], and the mxFold2 tool [35] was further utilized to predict the secondary structures of both the guide and passenger strands of siRNA. Additionally, DuplexFold [36] was employed to calculate the binding free energy between the guide strand and the target site.

### 2.2. Assembly of siRNA Nanoparticles

RNA motifs including siRNA sequences and nanoparticle assembly models were designed and predicted using RNAfold [37]. The assembly of nanoparticles was achieved by mixing equal amounts of designed RNA sequences (either purchased or obtained by transcription of PCR-amplified DNA templates) in a 20 nM concentration of MgSO_4_ solution, followed by incubation at 72 °C for 1 h.

### 2.3. Transmission Electron Microscopy (TEM) and Dynamic Light Scattering (DLS)

For transmission electron microscopy (TEM) analysis, 10 µL of the assembled siRNA nanoparticles was placed on a fresh glow-discharged continuous carbon-coated cropper grid, followed by phosphotungstic acid negative stain using standard drop method. The sample was then examined in JEM-1400Flash at 80 kV. For dynamic light scattering (DLS) analysis, 1 mL of a 10-fold diluted siRNA nanoparticles solution was evaluated using a Malvern ZSU3200 at 25 °C to determine the hydrodynamic diameter and its distribution.

### 2.4. Cell Cultures

Caco-2-N cells, expressing the N protein of SARS-CoV-2 [38], were gifted by Dr. Qiang Ding at Tsinghua University. Cells were cultured in DMEM-F12 medium supplemented with 10% fetal bovine serum (GIBCO), 50 units/mL of penicillin, 50 μg/mL of streptomycin (P/S, GIBCO), and 10 µg/mL of Blasticidin S hydrochloride (Solarbio) and incubated at 37 °C in a 5% CO_2_ incubator.

Vero cells were cultured in DMEM-F12 medium supplemented with 10% fetal bovine serum and 50 μg/mL penicillin/streptomycin (P/S) and incubated at 37 °C in a 5% CO_2_ incubator.

### 2.5. SARS-CoV-2 GFP/ΔN trVLP Production

The production of SARS-CoV-2 GFP/ΔN trVLP at the BSL-2 laboratory has been described previously [38]. This system was generously provided by Dr. Qiang Ding at Tsinghua University. Briefly, cDNA fragments of SARS-CoV-2 GFP/ΔN were synthesized and assembled into full-length cDNA, in which the N gene was replaced with the gene of the green fluorescent protein (GFP). RNA transcripts were produced by in vitro transcription using the mMESSAGE mMACHINE T7 Transcription Kit (ThermoFisher Scientific) and transfected into Caco-2-N cells by electroporation. The titer of the produced SARS-CoV-2 trVLP in the medium was titrated, and progeny trVLP was further amplified in Caco-2-N cells. All experiments were conducted in a BSL-2 laboratory.

### 2.6. SARS-CoV-2 GFP/ΔN trVLP Infection

Caco-2-N cells were seeded in a 12-well plate 24 h before infection. The cells were then infected with SARS-CoV-2 GFP/ΔN trVLP at a multiplicity of infection (MOI) of 0.05 and harvested 24 or 48 h post-infection. siRNA nanoparticles were added to the culture media at the indicated concentrations 24 h after infection. Inhibitors of cellular endocytosis, Pitstop2 (0.5 μM), Dynasore (40 μM), and MβCD (0.5 mM), were added to the culture media 24 h after infection for 25 min, after which the media were replaced with fresh media. All experiments with SARS-CoV-2 GFP/ΔN trVLP were conducted in a BSL-2 laboratory.

### 2.7. Confocal Microscopy

Vero cells were seeded into 3.5 cm glass-bottom dishes 24 h before treatment. After incubation with Cy5-labeled siRNA nanoparticles for 24 h, cells were fixed with 4% paraformaldehyde for 10 min, permeabilized in PBS containing 0.1% Triton X-100, and blocked with PBS containing 5% bovine serum albumin for 1 h. The fixed cells were subsequently incubated individually with primary antibodies against Lamp1 (CST, 9091s), Rab5a (CST, 2143T), and Rab7 (CST, 9367S) overnight at 4 °C, followed by incubation with fluorescent-labeled (Alexa Fluor 488) secondary antibodies for 2 h at room temperature. After washing the cells three times, a mounting medium with DAPI was applied, and images were acquired using a Nikon Ti2 confocal microscope.

### 2.8. RNA Extraction and RT-qPCR

Total cellular RNA was extracted using TRIzol reagent (Thermo). Reverse transcription quantitative PCR (RT-qPCR) was performed to assess the RNA level of SARS-CoV-2. In brief, 1 μg of total RNA was reverse transcribed using Hifair III 1st Strand cDNA Synthesis SuperMix for qPCR (Yeasen) to generate cDNA. qPCR was conducted using the 2X SYBR Green qPCR Mix (GDSBio) following the manufacturer’s instructions. The primers for viral RNA were as follows: V-F (5′-CGAAAGGTAAGATGGAGAGCC-3′) and V-R (5′-TGTTGACGTGCCTCTGATAAG-3′), which could amplify the segment of the ORF1ab gene of SARS-CoV-2. The primers for GAPDH were as follows: GAPDH-F (5′-GAAGGTGAAGGTCGGAGTC-3′) and GAPDH-R (5′-GAAGATGGTGATGGGATTTC-3′). The relative expression levels of the target genes were calculated using the comparative cycle threshold (CT) method, and all data were normalized relative to the housekeeping gene GAPDH.

### 2.9. Statistical Analysis

Statistical analyses were conducted using a two-tailed, unpaired Student’s *t*-test or one-way ANOVA with GraphPad Prism software (version 10.2.0). The results were presented as mean values with standard errors of the mean (SEMs) based on data from either two or three separate experiments.

## 3. Results

### 3.1. Design and Verification of siRNAs Targeting Highly Conserved Regions of SARS-CoV-2

To obtain siRNA sequences targeting the highly conserved regions of SARS-CoV-2, a systematic screen approach was employed, as illustrated in Figure 1. A pool of siRNA comprising 926 sequences, each 21 nucleotides in length, was generated via prediction using the siDirect2.0 tool. This prediction was based on the reference of the SARS-CoV-2 genome, adhering to Ui-Tei Rules, Reynolds Rules, or Amarzguioui Rules (URA Rules) to ensure that the melting temperature (Tm) of both strands was less than or equal to 21.5 °C and that the GC content ranged from 30% to 65%. This siRNA pool was narrowed down to 783 sequences with a coverage rate of over 98.5% of 153,345 SARS-CoV-2 genome sequences, encompassing major variants of concern (VOCs) including Alpha (B.1.1.7), Beta (B.1.351), Gamma (P.1), Delta (B.1.617.2), and Omicron (BA.5). The siRNA sequences targeting mRNAs with a high propensity for secondary structure formation were further excluded, resulting in a pool of 553 siRNAs. Off-target analysis was further conducted by aligning with the human genome GRCh38. Subsequently, the resulting 127 sequences underwent additional refinement through secondary structure toxicity and binding free energy analysis. This process ultimately yielded 18 siRNA sequences.

**Figure 2:**
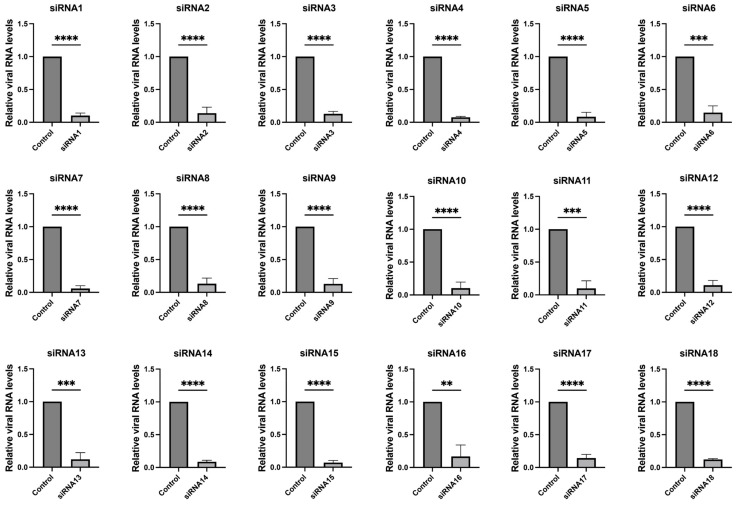
Inhibition of SARS-CoV-2 replication by siRNAs. Caco-2-N cells were infected with SARS-CoV-2 GFP/ΔN trVLP at an MOI of 0.05 and transfected with siRNAs (60 nM) 24 h post-infection, with a transfection efficiency of approximately 80% (Supplementary Figure S1). RNA was extracted and evaluated by RT-qPCR analysis (2^−ΔΔCT^) 48 h after treatment. The siRNA with a scrambled sequence, abbreviated as “Control”, served as the negative control in the experiment. Data were presented relative to the control treatment (set as 1) and were depicted as the mean ± standard error of the mean (SEM) from three independent experiments. Statistical significance is denoted as **** *p* < 0.0001, *** *p* < 0.001, ** *p* < 0.01 (Student’s *t*-test).

These 18 siRNAs target highly conserved genomic regions of SARS-CoV-2, encompassing both structural and non-structural proteins such as nucleocapsid, RNA-dependent RNA polymerase (RdRp), spike, NSP1, NSP3, NSP13, NSP14, NSP15, and NSP5. Details of the siRNA sequences and additional information are provided in Table 1. We further validated the efficacy of the screened siRNAs in the SARS-CoV-2 GFP/ΔN trVLP cell infection model. The results, depicted in Figure 2 and Supplementary Figure S2, demonstrated that all 18 siRNAs efficiently inhibited viral replication.

### 3.2. Construction and Characterization of siRNA Nanoparticles

To construct siRNA nanoparticles, the siRNA sequences were designed as RNA modules along with supportive RNA sequences. Subsequently, mixtures containing three siRNA motifs targeting spike-S2, NSP1_N, and NSP1_C were subjected to a mixing reaction to form siRNA nanoparticles, named siRNA_nano_1. Another type of siRNA nanoparticle, siRNA_nano_2, consisting of siRNAs targeting NSP15_middle domain, NSP3_single-stranded poly(A) binding domain, and NSP13_stem, was also constructed (Table 2).

**Figure 3:**
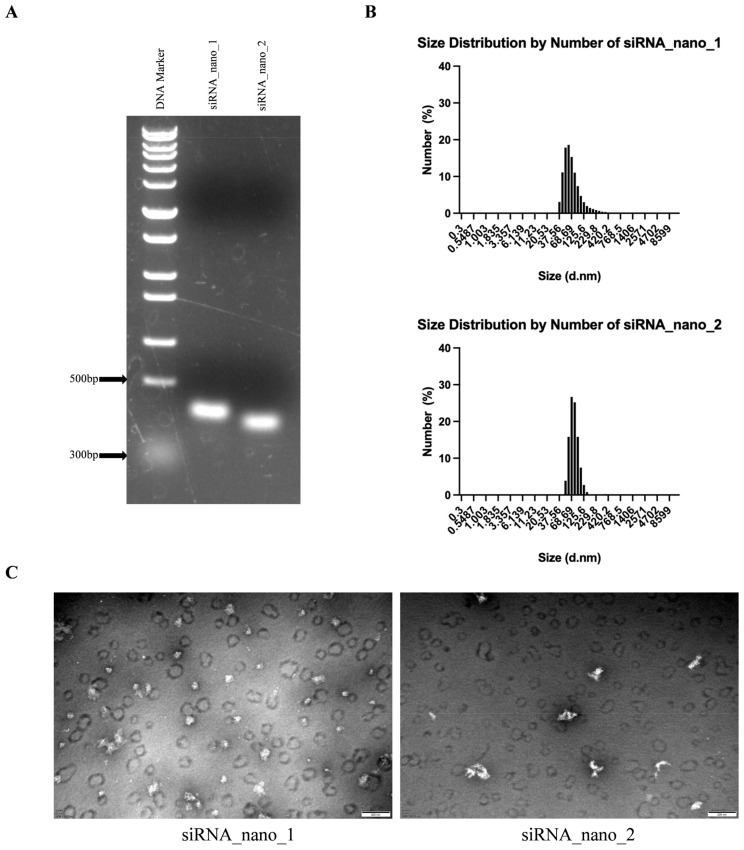
Characterization of siRNA nanoparticles. (**A**) Analysis of siRNA nanoparticles using native agarose gel electrophoresis. (**B**) Determination of the size distributions of siRNA nanoparticles using dynamic light scattering (DLS). (**C**) Visualization of siRNA nanoparticles using transmission electron microscopy (TEM). The images were captured at a magnification of 80,000×, and the scale bars in the micrographs measure 200 nm.

The self-assembled siRNA nanoparticles were then verified by native agarose gel electrophoresis. As shown in Figure 3A, the migration positions of both siRNA_nano_1 and siRNA_nano_2 on the gel were between 300 and 500 bp DNA, in accordance with the anticipated structure of siRNA nanoparticles. Further characterization of the nanoparticles was performed using a nanoparticle size analyzer. As depicted in Figure 3B, both types of siRNA nanoparticles exhibited good uniformity in size distribution, ranging from 50 to 100 nm. Transmission electron microscopy (TEM) results revealed that the nanoparticles exhibited closely intertwined chain-like structures, with sizes around 100 nm, as depicted in Figure 3C. These findings indicated the successful assembly of siRNA nanoparticles with excellent uniformity.

### 3.3. siRNA Nanoparticles Efficiently Inhibited SARS-CoV-2 Replication

**Figure 4:**
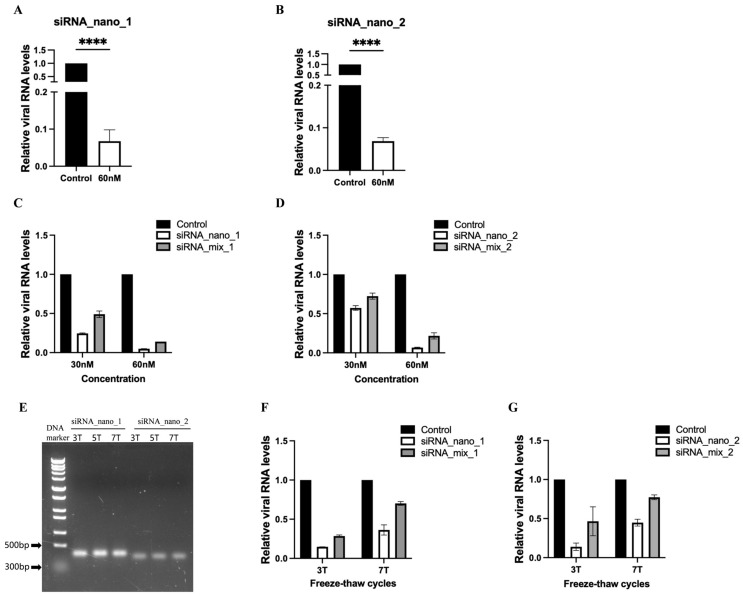
Inhibition of SARS-CoV-2 replication by siRNA nanoparticles. (**A**,**B**) Caco-2-N cells were infected with SARS-CoV-2 GFP/ΔN trVLP and treated with siRNA nanoparticles (60 nM) 24 h post-infection. At 48 h after treatment, RNA was extracted and evaluated by RT-qPCR analysis (2^−ΔΔCT^). (**C**,**D**) SARS-CoV-2 GFP/ΔN trVLP-infected Caco-2-N cells were treated with siRNA nanoparticles or transfected with a mixture of the same free siRNAs in the presence of liposomes at equivalent concentrations (30 nM and 60 nM) for 48 h. Subsequently, viral RNA was extracted and evaluated by RT-qPCR analysis (2^−ΔΔCT^). (**E**) Analysis of siRNA nanoparticles subjected to freeze–thaw treatment for indicated times using native agarose gel electrophoresis. (**F**,**G**) After undergoing freeze–thaw cycles either 3 or 7 times, siRNA nanoparticles or a mixture of the same free siRNAs at equivalent concentrations (50 nM) were administered to SARS-CoV-2 GFP/ΔN trVLP-infected Caco-2-N cells for 48 h, followed by extraction of viral RNA for evaluation via RT-qPCR analysis (2^−ΔΔCT^). The siRNA with a scrambled sequence, abbreviated as “Control”, served as the negative control in these experiments. Data were presented relative to the control treatment (set as 1) and are depicted as the mean ± standard error of the mean (SEM) from three (**A**,**B**) or two (**C**,**D**,**F**,**G**) independent experiments. Statistical significance is denoted as **** *p* < 0.0001 (Student’s *t*-test).

To assess the inhibitory effects of siRNA nanoparticles on SARS-CoV-2 replication, the nanoparticles were introduced into SARS-CoV-2 GFP/ΔN trVLP-infected Caco-2-N cells, and viral RNA levels were measured. The results indicated that both types of siRNA nanoparticles effectively inhibited virus replication, leading to a reduction of approximately 90% in viral RNA levels (Figure 4A,B).

Furthermore, we conducted a comparative analysis between the efficacy of siRNA nanoparticles and that of free siRNA (unassembled 21-nt siRNA) by incorporating a mixture of free siRNAs with the same sequences at identical concentrations in the inhibitory assay (Figure 4C,D and Supplementary Figure S3). The results demonstrated that, at equivalent concentrations, siRNA nanoparticles exhibited superior inhibitory effects compared to free siRNA, highlighting the enhanced efficacy of siRNA nanoparticles over free siRNA transfection.

We also assessed the stability of siRNA nanoparticles using freeze–thaw assays. The results indicated that minimal to no degradation of the nanoparticles was observed, even after subjecting them to seven cycles of freeze–thaw, as evidenced by agarose gel electrophoresis (Figure 4E). Although the inhibitory effect of siRNA nanoparticles against the virus showed a slight decline with an increase in freeze–thaw cycles, probably due to the partial disruption of its structural integrity, it remained more efficient than free siRNA (Figure 4F,G). Notably, after seven freeze–thaw cycles, siRNA nanoparticles still achieved approximately a 60% reduction in viral RNA levels, whereas free siRNA only resulted in a 30% reduction, indicating that free siRNA is more susceptible to the freezing–thawing process.

### 3.4. siRNA Nanoparticles Enter Cells Directly through Cellular Endocytic Pathways

It has been reported that the primary route of entry for nanoparticles into cells is via endocytosis [39]. To assess whether siRNA nanoparticles also utilize cellular endocytosis for cell entry, siRNA nanoparticles labeled with Cy5 were incubated with Caco-2-N cells infected with SARS-CoV-2 GFP/ΔN trVLP in the presence of inhibitors against clathrin-mediated endocytosis (Pitstop 2), dynamin-mediated endocytosis (Dynasore), or cholesterol-mediated endocytosis (MβCD), respectively.

**Figure 5:**
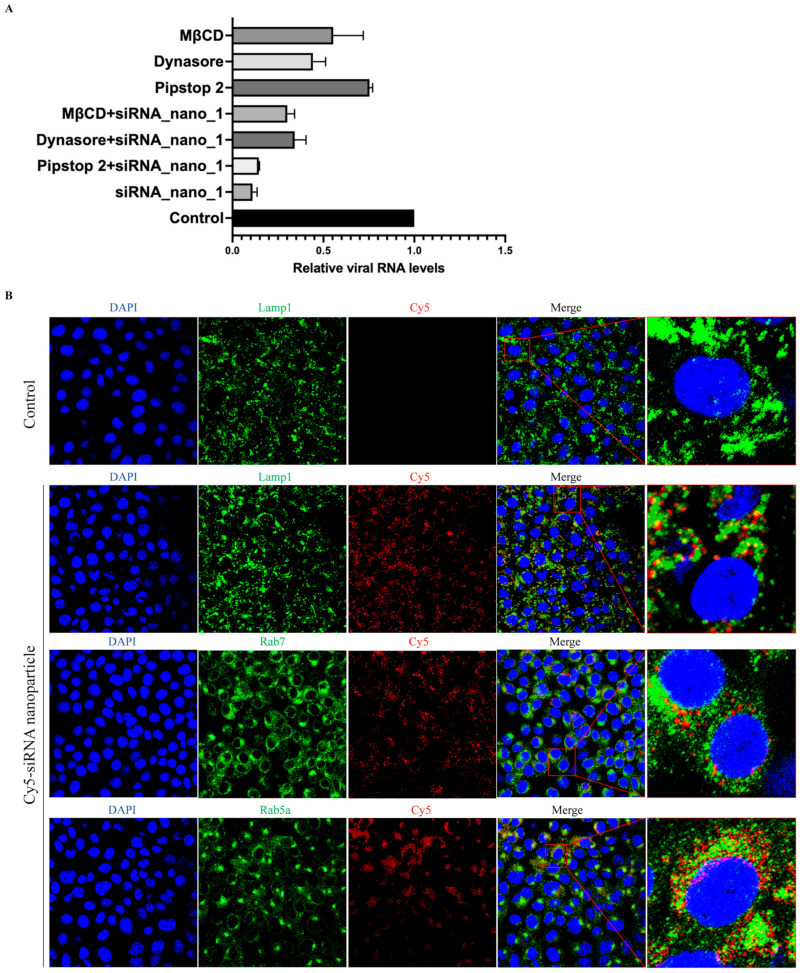
siRNA nanoparticles enter cells via endocytosis. (**A**) SARS-CoV-2 GFP/ΔN trVLP-infected Caco-2-N cells were treated with siRNA nanoparticles alone (60 nM) for 48 h, or with cellular endocytosis inhibitors alone (0.5 μM of Pitstop2, or 40 μM of Dynasore, or 0.5 mM of MβCD) for 25 min, or with both siRNA nanoparticles and inhibitors at the same concentrations for the same time. Viral RNA was then extracted and evaluated by RT-qPCR analysis (2^−ΔΔCT^). The siRNA with a scrambled sequence, abbreviated as “Control”, served as the negative control in these experiments. Data were presented relative to the control treatment (set at 1) and represented the mean ± standard error of the mean (SEM) from two independent experiments. (**B**) After incubation with Cy5-labeled siRNA nanoparticles for 24 h, Vero cells were then fixed and incubated with antibodies against Lamp1, Rab7, and Rab5a, respectively, followed by being stained with Alexa Fluor 488-conjugated secondary antibodies. The nuclei were stained with DAPI and the subcellular localization of siRNA nanoparticles was visualized with a confocal fluorescence microscope.

The results showed that, as depicted in Figure 5A, these endocytosis inhibitors exhibited inhibitory effects on viral infection. This finding was consistent with previous studies demonstrating that SARS-CoV-2 utilizes endocytosis for cellular entry and inhibitors targeting these pathways can impede virus entry [40,41]. Interestingly, combined treatment with both siRNA nanoparticles and inhibitors did not result in an enhanced inhibitory effect on viral replication compared to siRNA nanoparticles alone. This suggested that the presence of endocytosis inhibitors primarily affected the entry of siRNA nanoparticles into cells rather than viral entry, thereby resulting in a reduced level of virus replication. These findings indicated the dependence of siRNA nanoparticle entry on cellular endocytic pathways.

To further validate the involvement of endocytosis in the uptake of siRNA nanoparticles, confocal fluorescence microscopy was employed to examine the subcellular localization of these nanoparticles. The results indicated a partial colocalization of siRNA nanoparticles with early endosomes (Rab5a), late endosomes (Rab7), and lysosomes (Lamp1) (Figure 5B). This observation further suggested that siRNA nanoparticles underwent cellular internalization directly through the cellular endocytic pathways.

## 4. Discussion

The development of vaccines, antiviral therapies, and immunomodulatory treatments has proven effective in combating COVID-19, reducing disease burden and mortality rates [15,42]. However, the rapid emergence of various variants during the pandemic has compromised the effectiveness of these interventions and led to resistance against vaccines and antiviral drugs [43]. There is an ongoing need to develop novel therapeutics capable of addressing current and future variants of SARS-CoV-2, which may contribute to future epidemics or pandemics. RNA interference (RNAi) technology offers a promising avenue to address this challenge, with several studies demonstrating its inhibitory effects on SARS-CoV-2 [11,13,14,16]. Nonetheless, RNAi technology also faces challenges, including the potential for viral mutations to confer drug resistance and concerns regarding the efficiency and safety of delivery methods [8,44].

In this study, we designed a set of siRNAs targeting the highly conserved regions of SARS-CoV-2, with the targeted genes showing conservations above 98.5% across all aligned VOCs, including Alpha (B.1.1.7), Beta (B.1.351), Gamma (P.1), Delta (B.1.617.2), and Omicron (BA.5), reaching up to 99.9% in some sequences. When initiating our research, we incorporated the most prevalent variants known at that time. With the emergence of new VOCs, such as the now-dominant JN.1 variant and its descendants, we further validated the potential inhibitory activities of the designed siRNAs against these new VOCs. These 18 siRNA sequences were aligned with 54,784 viral gene sequences recorded between January 1, 2024 and May 12, 2024, primarily comprising the JN.1 variant and its sublineage JN.1.4. The results indicated that all siRNAs maintained high coverage rates above 88%, with 10 siRNAs exceeding 95%. We further conducted additional alignment of these siRNAs with 32,529 published JN.1 sequences. The results revealed that all siRNAs exhibited coverage rates of at least 89%, with 13 siRNAs exceeding 95% coverage. These results offer reassurance regarding the sustained effectiveness of the siRNAs against the prevailing strains of SARS-CoV-2, encompassing the JN.1 variant and its sublineages.

The siRNAs demonstrated significant inhibitory effects against SARS-CoV-2 with the reference genome. We further developed siRNA nanoparticles targeting key regions of the SARS-CoV-2 genome, including NSP1, NSP3, NSP13, NSP15, and spike_S2, which are crucial for viral replication based on their known functions [45,46,47,48]. Our findings confirmed the efficacy and efficiency of these siRNA nanoparticles in inhibiting SARS-CoV-2 replication.

Given the high conservation of the target regions, it is theoretically plausible that the siRNAs in our pool could effectively suppress the replication of other variants of concern of SARS-CoV-2, even including potential future variants. Furthermore, a single nanoparticle in this study can encapsulate up to 12 siRNA sequences, allowing for the construction of siRNA nanoparticles that concurrently target multiple viral genes or different target sites within the same gene, thus enhancing the antiviral effectiveness and also restricting the development of escape mutations of the virus. Further studies are warranted to address the broad-spectrum activities of siRNA nanoparticles against various VOCs of SARS-CoV-2, particularly when utilizing a wider range of combinations of multiple siRNA sequences.

Meanwhile, siRNAs targeting relatively conserved genes in human coronavirus (HCoV), including SARS-CoV, MERS-CoV, SARS-CoV-2, HCoV-OC43, and HCoV-HKU1, can be further screened and designed to construct siRNA nanoparticles with broad-spectrum anti-HCoV activities. Alternatively, mosaic siRNA nanoparticles targeting multiple viruses could also be developed. The development of broad-spectrum antivirals against HCoVs based on siRNA nanoparticles offers a promising approach for treating current viral infections and potentially emerging ones in the future.

In this study, siRNA nanoparticles exhibited remarkable stability, maintaining structural integrity even after undergoing multiple freeze–thaw cycles while retaining their efficacy in suppressing viral replication. This suggests that they can be stored and transported at room temperature for short durations. Furthermore, the enhancements in the stability of RNA nanoparticles can be achieved through further structural optimization and chemical modifications, which could significantly enhance the resistance to degradation and thermal stability, thereby maintaining their functionality in non-cold and fluctuating temperature environments [49,50,51].

Furthermore, our findings revealed that these siRNA nanoparticles can enter cells via endocytic pathways without the need for additional delivery carriers. They exhibited remarkably high internalization efficiency, with up to 95% of cells showing the presence of nanoparticles after 24 h of incubation (Supplementary Figure S4). This nucleic acid nanotechnology-based siRNA delivery approach not only enhances delivery efficiency but also mitigates the potential risks of cytotoxicity and adverse immunogenic reactions commonly associated with carrier-based delivery systems [17,50,51,52].

The characteristics of siRNA nanoparticles, such as their high stability, efficient cellular uptake, and biocompatibility, render them adaptable to diverse routes of administration, encompassing nasal and oral delivery modalities. Specifically, nasal delivery offers high effectiveness and convenience for treating respiratory pathogens by directly targeting the early infection site, leading to a substantial increase in local drug concentration while minimizing systemic side effects [53,54]. These advantages significantly broaden the potential of siRNA therapies in clinical applications.

In summary, in this study, we designed siRNAs targeting highly conserved regions of SARS-CoV-2 and constructed siRNA nanoparticles utilizing RNA self-assembly technology. These nanoparticles can be efficiently internalized by cells via endocytic pathways and effectively inhibit viral replication. Moreover, the unique characteristics of the siRNA nanoparticles, including their stability, programmability, modifiability, and ease of preparation, offer promising avenues for the development of broad-spectrum antiviral drugs against viral infections beyond COVID-19.

## Figures and Tables

**Table 1:** Information of designed siRNAs.

ID	Strand	Sequence (5′-3′)	Secondary Structure	Coverage	Target Genes
siRNA1	guide	UGUGUUUUCUCGUUGAAACCA	((.(((((......))))))) (−0.8)	99.83%	NSP1_N
	passenger	GUUUCAACGAGAAAACACACG	(((((.....)))).)..... (−3.3)		
siRNA2	guide	UAUUACCGUUCUUACGAAGAA	..((..(((....)))..)). (−2)	99.69%	NSP1_C
	passenger	CUUCGUAAGAACGGUAAUAAA	((((....))).)........ (−2.3)		
siRNA3	guide	UAUUAUUGGGUAAACCUUGGG	(......((.....))....) (−0.5)	99.36%	Nucleocapsid
	passenger	CAAGGUUUACCCAAUAAUACU	..((..(((.....)))..)) (−4)		
siRNA4	guide	UUGUAUAUGCGAAAAGUGCAU	.(((((.........))))). (−0.1)	99.52%	RdRp
	passenger	GCACUUUUCGCAUAUACAAAA	((.......)).......... (−2.9)		
siRNA5	guide	AAUAAACACGCCAAGUAGGAG	..........((.....)).. (−1.8)	99.90%	Spike_S2
	passenger	CCUACUUGGCGUGUUUAUUCU	((.....))...(......). (−2.8)		
siRNA6	guide	GAAUUCCAAGCUAUAACGCAG	.........((......)).. (−3.9)	99.69%	Spike_RBD
	passenger	GCGUUAUAGCUUGGAAUUCUA	((......))........... (−2.3)		
siRNA7	guide	UUUCAACGUACACUUUGUUUC	....((((.......)))).. (−4.7)	99.77%	Spike_NTD
	passenger	AACAAAGUGUACGUUGAAAUC	............(.......) (−4.9)		
siRNA8	guide	GUAGCUUUGAGCGUUUCUGCU	((((............)))). (−0.5)	99.77%	NSP13_Stem
	passenger	CAGAAACGCUCAAAGCUACUG	(((.(..(((...)))).))) (−0.8)		
siRNA9	guide	UUAGUUACACGAUAACCAGUA	(..((((.....))))....) (−2.8)	99.93%	NSP13_1B
	passenger	CUGGUUAUCGUGUAACUAAAA	.(((((((...)))))))... (−0.7)		
siRNA10	guide	ACAUCAUGCGUGAUAACACCC	..((((....))))....... (−2.6)	98.89%	NSP13_helicase
	passenger	GUGUUAUCACGCAUGAUGUUU	(...(((((....)))))..) (−0.6)		
siRNA11	guide	UAAGAAUGGUCUACGUAUGCA	(..(.(((.....))).)..) (−2.7)	99.46%	NSP13_ZBD
	passenger	CAUACGUAGACCAUUCUUAUG	((((...(((....))))))) (−0.4)		
siRNA12	guide	AGCUUUAGGGUUACCAAUGUC	.((....((....))...)). (−0.5)	99.87%	NSP14
	passenger	CAUUGGUAACCCUAAAGCUAU	...((((.........)))). (−3.3)		
siRNA13	guide	UAAACGAUAUGUUCGAAGGCA	....(((.....)))...... (−2.5)	99.79%	NSP15_NendoU domain
	passenger	CCUUCGAACAUAUCGUUUAUG	(...(((.....))).....) (−1.8)		
siRNA14	guide	UCUACUUGACCAUCAACUCUA	(....((((...))))....) (−3)	99.66%	NSP15_Middle domain
	passenger	GAGUUGAUGGUCAAGUAGACU	.((((.((......)).)))) (−2.3)		
siRNA15	guide	AAAAUCUAGCACCAUAAUCAA	..................... (−1.9)	99.85%	NSP3 _Single-stranded poly(A) binding domain
	passenger	GAUUAUGGUGCUAGAUUUUAC	(...((........))....) (−3.5)		
siRNA16	guide	ACAAACACGGUUUAAACACCG	.......((((......)))) (−2)	99.76%	NSP3 _PLpro
	passenger	GUGUUUAAACCGUGUUUGUAC	(((..(((((...)))))))) (−0.1)		
siRNA17	guide	AAUUACAACCGUCUACAACAU	..................... (−2.9)	99.84%	NSP3_C
	passenger	GUUGUAGACGGUUGUAAUUCA	(...((.(....).))...). (−3.3)		
siRNA18	guide	GUAAACUACGUCAUCAAGCCA	..................... (−2.6)	99.54%	NSP5
	passenger	GCUUGAUGACGUAGUUUACUG	(........).(((....))) (−3.1)		

The “Secondary structure” column represents RNA secondary structures using dot–bracket notation. Unpaired nucleotides are indicated by dots (.), while base pairs are depicted using matching parentheses. Specifically, “(“ denotes the 5′ end of a pair, and “)” denotes the 3′ end.

**Table 2:** Construction of siRNA nanoparticles.

siRNA Nanoparticles	siRNA	RNA Motif	Length	Target Gene
siRNA_nano_1	siRNA1	CUCCUACUUGGCGUGUUUAUUCCUGUCAAUCAUGGCAAGUGUGUUUUCUCGUUGAAACCA	60	NSP1_N
	siRNA2	UGGUUUCAACGAGAAAACACACUUGUCAUGUGUAUGUUGCCUUAUUACCGUUCUUACGAAGAA	63	NSP1_C
	siRNA5	UUCUUCGUAAGAACGGUAAUAAGGCACAUACUUUGUUGAUAGGAAUAAACACGCCAAGUAGGAG	64	Spike_S2
siRNA_nano_2	siRNA8	AGCAGAGACGCUCGGAGCUGCUGUUUUGGUCUACUUGACCAUCAACUCUA	50	NSP15_Middle domain
	siRNA14	UAGAGUUGGUGGUCGAGUAGGCCUUUUGCAAAAUCUAGCACCAUAAUCAA	50	NSP3 _Single-stranded poly(A) binding domain
	siRNA15	UUGAUUAUGGUGCUAGGUUUUGCUUUUCAGUAGCUUUGAGCGUUUCUGCU	50	NSP13_Stem

## Data Availability

All of the data are present in the manuscript.

## Supplementary Materials

The following supporting information can be downloaded at: https://www.mdpi.com/article/10.3390/v16071072/s1, Figure S1: Cellular uptake of FAM-labeled siRNA; Figure S2: Inhibition of SARS-CoV-2 infection by siRNAs; Figure S3: Inhibition of SARS-CoV-2 infection by siRNA nanoparticles; Figure S4: Internalization of Cy5-siRNA nanoparticles.
