# Supplementary material for: Inhibition of SARS-CoV-2 Replication by Self-Assembled siRNA Nanoparticles Targeting Multiple Highly Conserved Viral Sequences
Source: Viruses. 2024 Jul 3;16(7):1072. doi: 10.3390/v16071072 (PMC11281333; doi:10.3390/v16071072)
Supplement: Supplementary file 1 [file viruses-16-01072-s001.zip › viruses-3010086-supplementary.pdf]

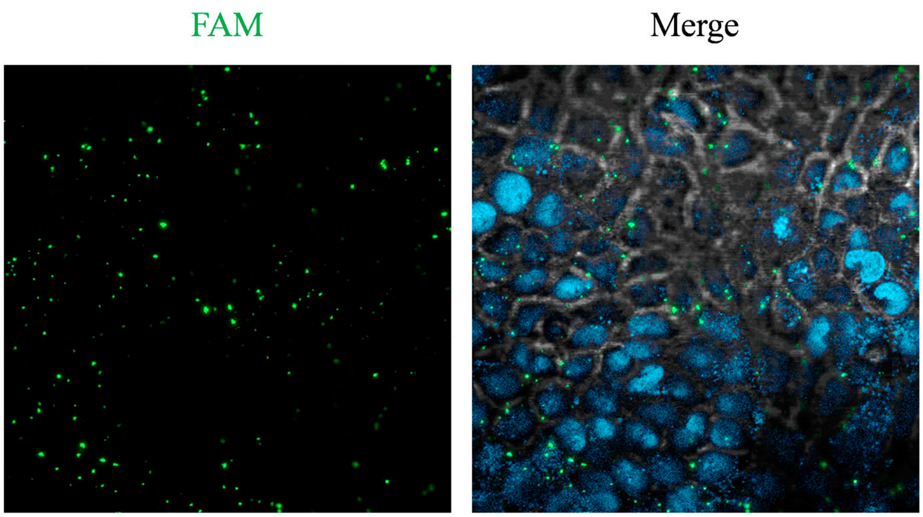

Supplementary Figure S1. Cellular uptake of FAM-labeled siRNA. Caco-2-N cells were transfected with 60 nM of FAM-labeled siRNA and were washed and fixed 12 hours post transfection. The cellular uptake of FAM-labeled siRNA was visualized using a fluorescence microscope.

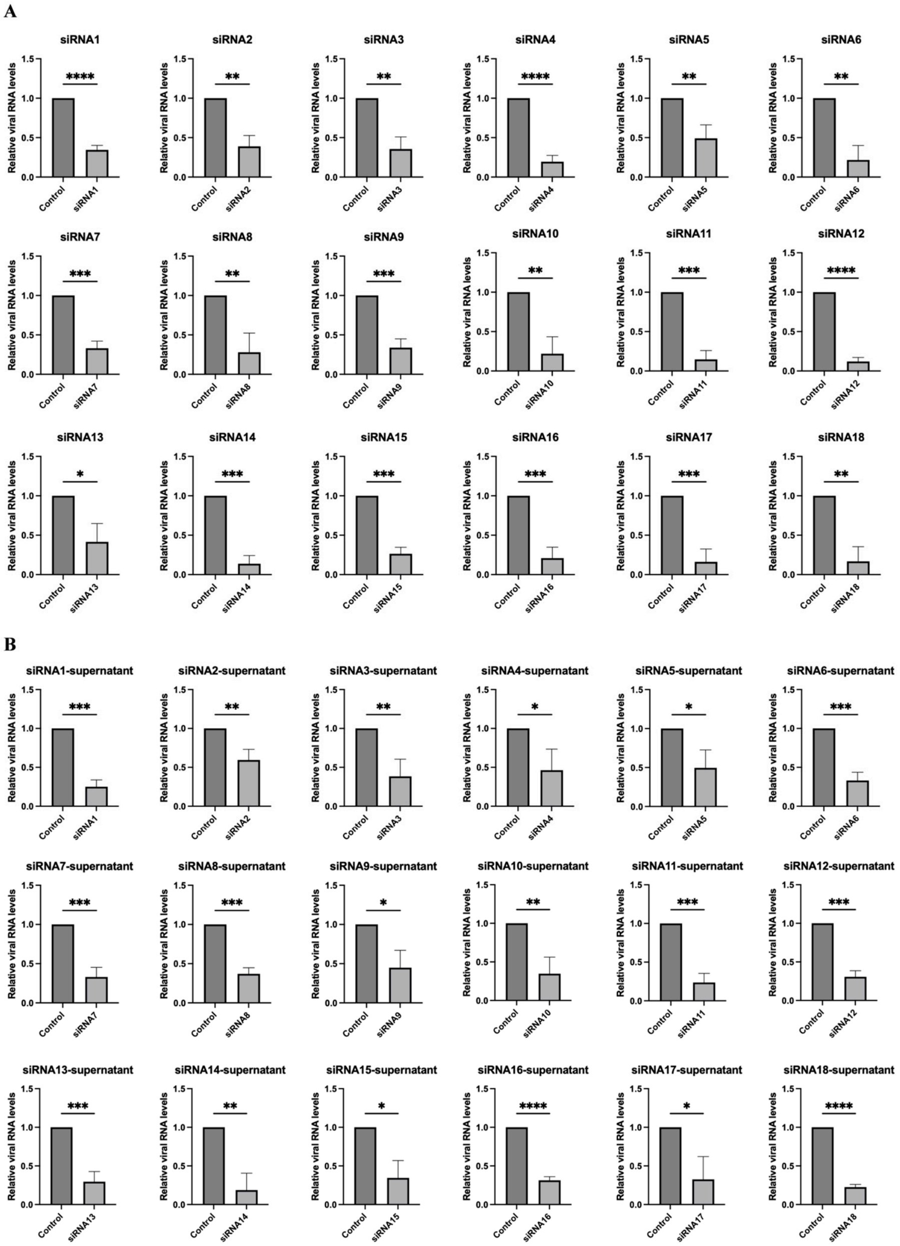

Supplementary Figure S2. Inhibition of SARS-CoV-2 infection by siRNAs. Caco-2-N cells were infected with SARS-CoV-2 GFP/ΔN trVLP at a MOI of 0.1 and transfected with siRNAs (30 nM) 24 hours post-infection. Intracellular viral RNA **(A)** and released viral RNA in the supernatant **(B)** were extracted 24 hours after treatment and evaluated by RT-qPCR analysis. Data were presented relative to the control treatment (set as 1) and were depicted as the mean ± standard error of the mean (SEM) from three independent experiments. Statistical significance is denoted as **** $p < 0.0001$, *** $p < 0.001$, ** $p < 0.01$, * $p < 0.05$ (Student's t-test).

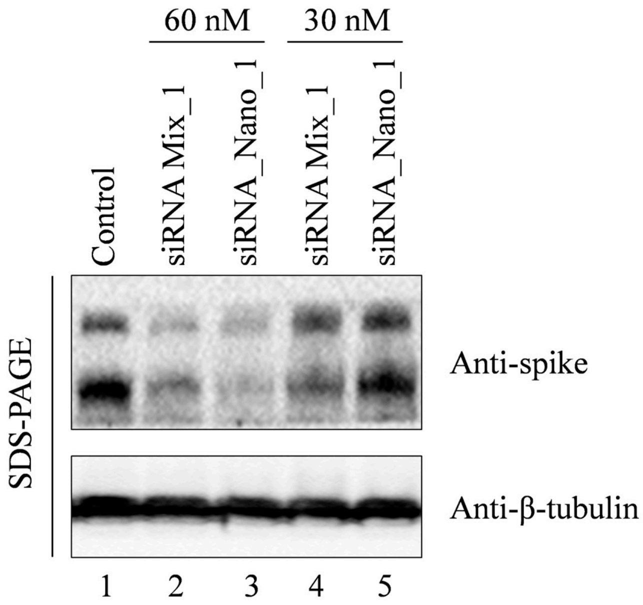

Supplementary Figure S3. Inhibition of SARS-CoV-2 infection by siRNA nanoparticles. Caco-2-N cells were infected with SARS-CoV-2 GFP/ΔN trVLP at a MOI of 0.1 and subsequently treated with siRNA nanoparticles or transfected with a mixture of the same free siRNAs at equivalent concentrations (30 nM and 60 nM) for 24 hours. Following treatment, cells were lysed, and the expression of viral spike protein was analyzed by SDS-PAGE and western blotting.

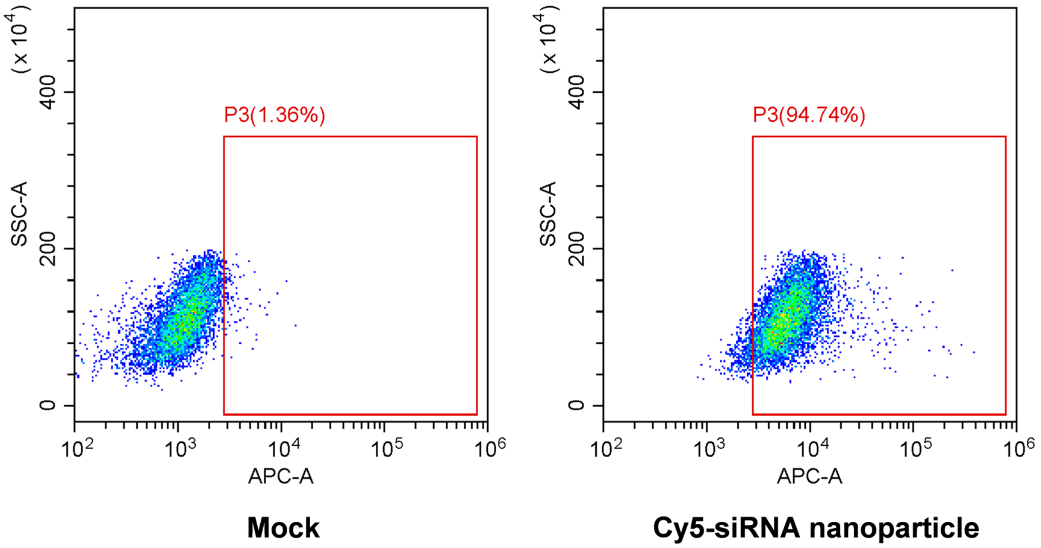

Supplementary Figure S4. Internalization of Cy5-siRNA nanoparticles. Vero cells were incubated with Cy5-labeled siRNA nanoparticles for 24 hours, followed by harvesting, treatment with trypsin, and washing with PBS three times. The internalization of siRNA nanoparticles was then detected using flow cytometry.
